# Morphological and functional characteristics of human gingival junctional epithelium

**DOI:** 10.1186/1472-6831-14-30

**Published:** 2014-04-03

**Authors:** Qian Jiang, Youcheng Yu, Hong Ruan, Yin Luo, Xuehua Guo

**Affiliations:** 1 Department of Stomatology, Zhongshan Hospital Fudan University, 180 Fenglin Road, Shanghai 200032, China

**Keywords:** Junctional epithelium, Oral gingival epithelium, Cytokeratin, Immunohistochemistry, Co-culture

## Abstract

**Background:**

This study aims to observe the morphological characteristics and identify the function characteristics of junctional epithelium (JE) tissues and cultured JE cells.

**Methods:**

Paraffin sections of human molar or premolar on the gingival buccolingual side were prepared from 6 subjects. HE staining and image analysis were performed to measure and compare the morphological difference among JE, oral gingival epithelium (OGE) and sulcular epithelium (SE). Immunohistochemistry was applied to detect the expression pattern of cytokeratin 5/6, 7, 8/18, 10/13, 16, 17, 19, and 20 in JE, OGE and SE. On the other hand, primary human JE and OGE cells were cultured in vitro. Cell identify was confirmed by histology and immunohistochemistry. In a co-culture model, TEM was used to observe the attachment formation between JE cells and tooth surface.

**Results:**

Human JE was a unique tissue which was different from SE and OGE in morphology. Similarly, morphology of JE cells was also particular compared with OGE cells cultured in vitro. In addition, JE cells had a longer incubation period than OGE cells. Different expression of several CKs illustrated JE was in a characteristic of low differentiation and high regeneration. After being co-cultured for 14 d, multiple cell layers, basement membrane-like and hemidesmosome-like structures were appeared at the junction of JE cell membrane and tooth surface.

**Conclusions:**

JE is a specially stratified epithelium with low differentiation and high regeneration ability in gingival tissue both in vivo and in vitro. In co-culture model, human JE cells can form basement membrane-like and hemidesmosome-like structures in about 2 weeks.

## Background

Gingival epithelium consists of three regions: oral gingival epithelium (OGE), sulcular epithelium (SE) and Junctional epithelium (JE). JE is a specialized gingival epithelium locating at the junction of periodontal soft tissue and hard tissue, and attaching to the crown or root like a collar. JE cells are uniform in shape (either flat or spindle) and aligned parallel to the tooth surface, containing large intercellular spaces due to relaxed cell junctions [1]. As a special structure at dento-gingival junction, JE is different from other epitheliums (OGE, SE) in origin, cell morphology, proliferation and differentiation [2,3]. Meanwhile, it has been reported that JE is critical to maintain the integrity of periodontal tissue [4,5] and is a key area for primary onset of periodontal diseases and treatments [6]. Besides, Neutrophil a-defensins was found to localize in the junctional epithelium, which has significant effects on the epithelial integrity and functioning (keratinocyte adhesion, spread, and proliferation), and the effects are beyond their antibacterial activities [7]. However, it is still unclear and controversial about JE in the differentiation, phagocytic activity, mechanism of its attachment to tooth surface, repair and reconstruction mechanism after injury [5,8].

The conventional histological methods for investigation of JE in vivo are simplistic in approach and limited in the range of observation [9-12]. In recent years, scholars have studied the JE using in vitro cell culture models and molecular cytological techniques using animal and/or human OGE cells, periodontal ligament epithelial cells and oral epithelial cells [13-16]. Though these cells are oral epithelial cells, they cannot model primary JE cells completely due to differences in source, morphology, structure, differentiation and stimuli that induce proliferation.

Cytokeratins (CKs) are intermediate filament proteins of cytoskeleton family and are the major structural proteins in epithelial cells. As we know, the expression of keratins is one of the definitive characteristics of epithelial cells and reflects the biological properties of epithelial cells, including their origination, development, histological type, and level of differentiation [17,18]. Several researches have studied the expression and distribution of a variety of CKs (CK-pan, 5/6, 7, 8/18, 10/13, 16, 17, 19, 20) in periodontal tissues of humans and animals, and the expression of some keratins in gingival epithelium were determined [15,19-21]. For example, the expression patterns of CK10/13, 16, 19 in JE were different from that in OGE and SE; The especially high expression of CK19 in all layers of JE made it became a characteristical histological marker for JE in vivo [3,22-24]. However, the expressions of various types of cytokeratin in JE and the difference with OGE and SE have not been systematically reported.

In this study, the morphological characteristics of JE tissues were examined by histological observation, image analysis and immunohistochemistry. The expression and distribution of a variety of CKs were determined in JE tissues and compared with OGE and SE. Besides, primary JE and OGE cells were cultured. The morphological structure and growth pattern of primary JE and OGE cells were observed and the expressions of specific keratins (CK-pan, 19, 10/13, 16) were also detected by immunohistochemistry. We suspect to identify the unique biological properties (morphology, regenerative potential) of JE in vivo and vitro. Furthermore, cultured human JE cells were seeded directly onto human root slices in a composite culture in order to explore the process of JE new attachment. This would provide experimental evidence for further study of how new attachment occurs after periodontal surgery and the formation of peri-implant tissue healing in clinic.

## Methods

### Morphological characteristics of human gingival epithelium tissues

Human gingival specimens were isolated from mandible specimens of four male and two female patients with mandibular ameloblastoma. They were non-smokers without any other diseases. The age and sampling site was list in Table 1. A ablative surgery was carried out in the department of oral pathology at the Ninth People's Hospital Affiliated to Shanghai Jiao Tong University between September and October of 2010. The study was approved by the local Ethics Committee, and informed consent was obtained from all patients. Mandibular molar or premolar and its gingival tissues distant from the tumor site with normal gingival morphology were selected by clinical observation. In addition, the samples less than 3 mm in depth detected by periodontal probing were collected and fixed in 10% formaldehyde at room temperature for 24 h. The samples were placed in Plank-Rychlo decalcifying solution (70 g of AlCl_3_, 56 ml of formic acid, 85 ml of hydrochloride acid, and distilled water added to 1 L) for 2 weeks after they were cut vertical to the long axis of the tooth along the buccolingual side using a hand saw. Finally, several 5 mm thick sections of tooth and gingival tissues in the center of the tooth were obtained from each specimen. Then these sections were subjected to ethanol dehydration and paraffin embedded. Three paraffin blocks were selected randomly from each specimen and serially sectioned at 4 μm. The slices were stained with hematoxylin-eosin (HE) and observed under light microscope. The width, thickness, area of JE and the width of SE on the buccal side were measured by Axioplan 2 image analysis system.

**Table 1:** Measured dimensions in human JE and SE

**Sample no.**	**Age**	**Sampling site**	**Width of JE (mm)**	**Thickness of JE (mm)**	**Area of JE (mm**^**2**^**)**	**Width of SE (mm)**
1	21	Premolar	1.135	0.069	0.047	0.325
			1.134	0.066	0.045	0.321
			1.133	0.064	0.043	0.334
2	28	Molar	1.113	0.062	0.043	0.308
			1.102	0.066	0.044	0.303
			1.120	0.065	0.044	0.323
3	31	Molar	0.850	0.058	0.041	0.563
			0.845	0.059	0.041	0.565
			0.847	0.060	0.041	0.554
4	28	Molar	0.838	0.052	0.038	0.550
			0.844	0.055	0.040	0.552
			0.858	0.056	0.040	0.543
5	33	Premolar	1.105	0.083	0.043	0.785
			1.108	0.083	0.043	0.801
			1.105	0.083	0.041	0.808
6	38	Premolar	1.081	0.069	0.041	0.625
			1.072	0.065	0.038	0.667
			1.078	0.069	0.040	0.644
Mean			1.021	0.066	0.042	0.532
Standard deviation			0.128	0.009	0.002	0.176

The sample 2, 3 were females and the left were males.

**Figure 1:**
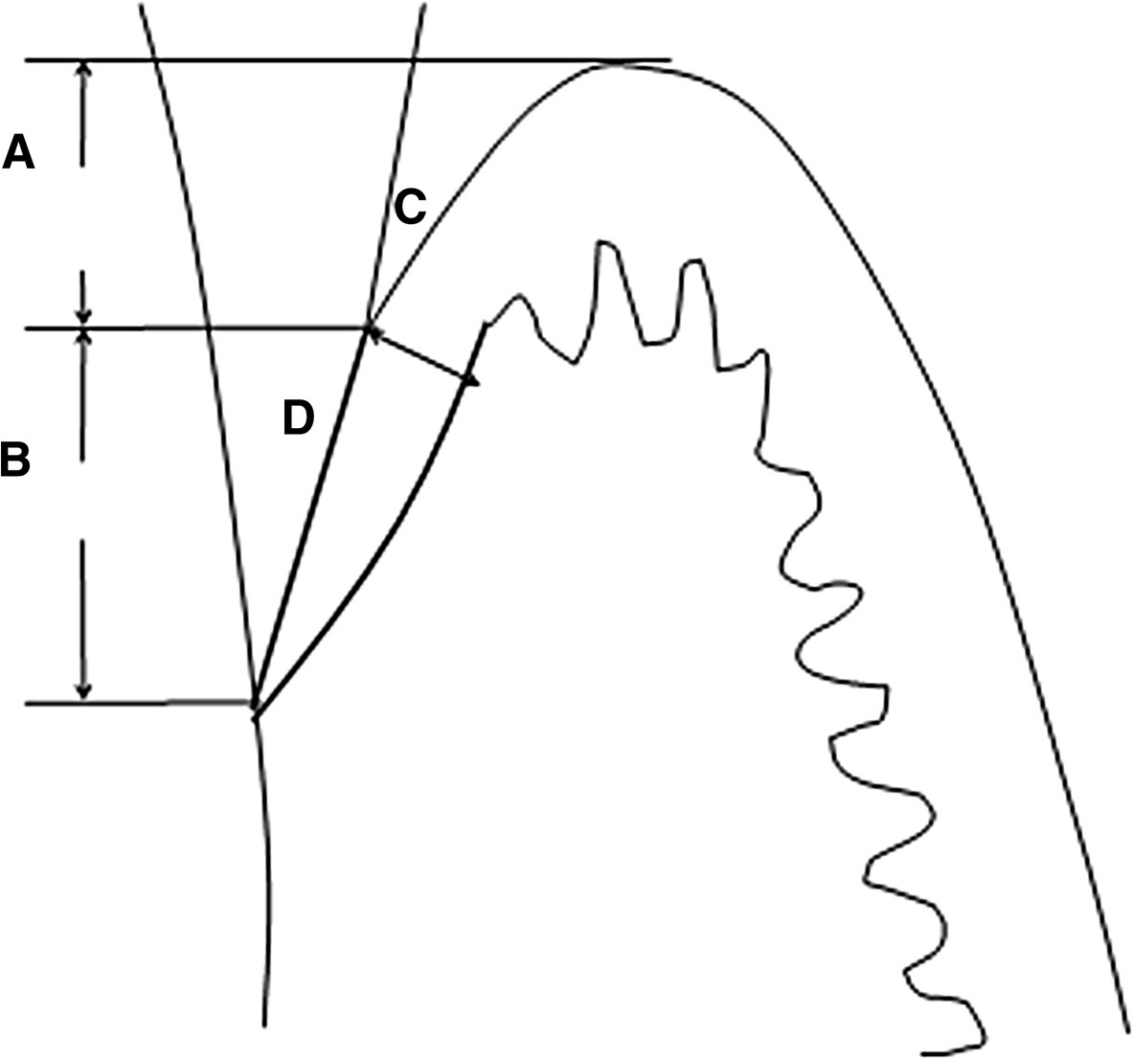
**Schematics description of measurement JE and SE. A**. SE width; **B**. JE width; **C**. JE thickness; **D**. JE area.

Due to separation of JE with the tooth surface after decalcification, the boundary between JE and SE was determined according to epithelial ridges, keratinization, cell morphology, and staining. According to the projection method, perpendicular lines were drawn from free margin of SE, junction of JE and SE, and the most root part of JE toward the tooth surface, respectively. The projection lengths of SE and JE on the tooth surface were measured as their widths. A perpendicular line was drawn at the thickest part of JE and the distance between the two intersection points of perpendicular line and epithelium was measured as the thickness of JE. A curve was drawn from the most root part of JE to its junction with SE including the entire JE region and area (Figure 1).

### Human JE and OGE cells culture

**Figure 2:**
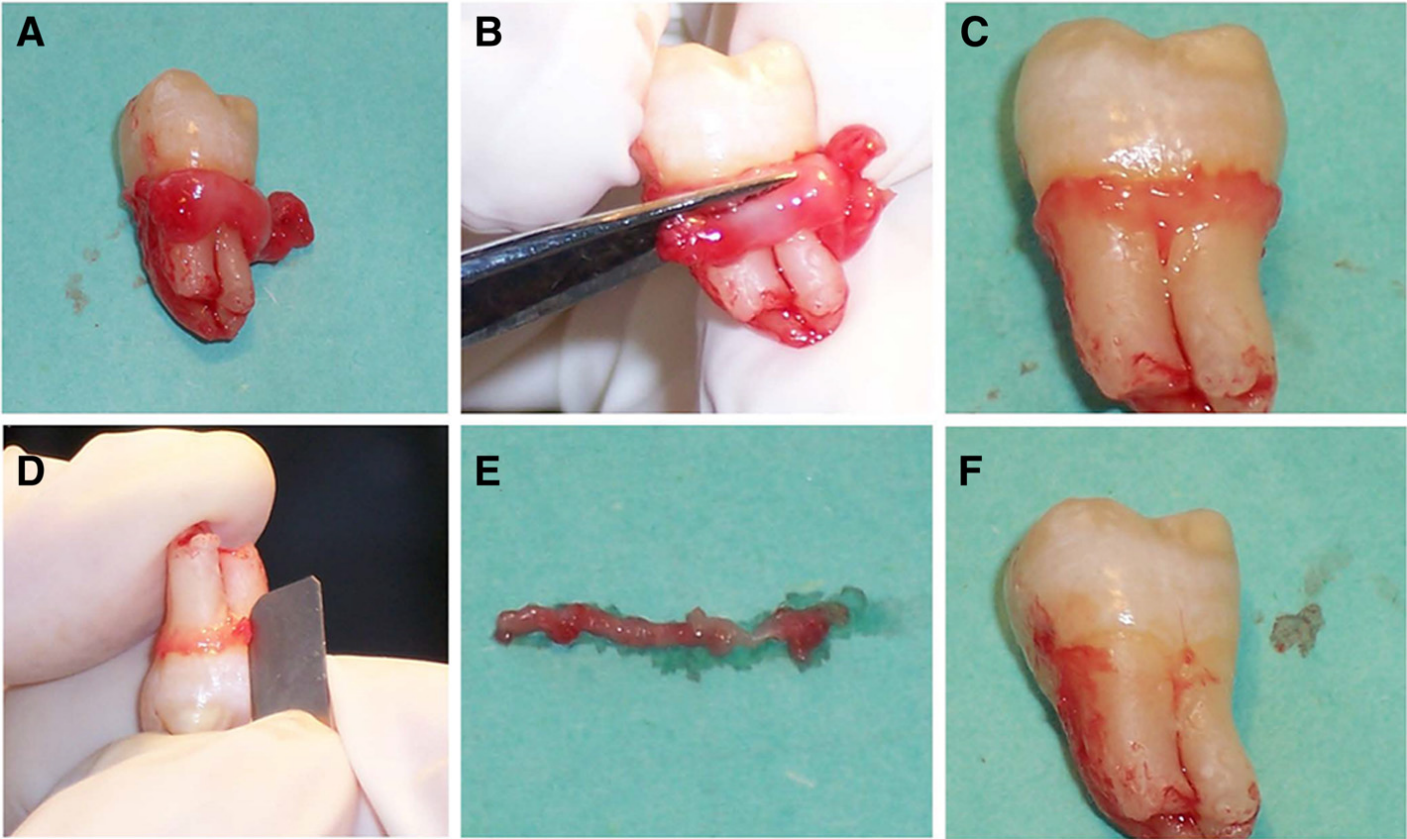
**JE samples. A**. Remove fully erupted impacted tooth with marginal gingival; **B**. Cut off the excess free gingiva (including OGE and SE); **C**. Remain JE tissue on the tooth neck; **D**. Obtain JE tissue by scraping on the tooth neck; **E**. Removed JE tissues; **F**. Smooth tooth neck after scraping.

In order to culture JE and OGE cells in vitro, incisions of 2 mm in length were made along buccal and lingual marginal gingivae. Five healthy and fully erupted teeth were removed along with orthodontic or impacted teeth (12 to 25 years old, good oral health and clinical healthy gingiva). The teeth together with the incised marginal gingiva were removed. The free gingiva (including OGE and SE) was cut off as much as possible macroscopically. JE tissues tightly attached to the tooth neck (not the root) were scraped off from the tooth surface (Figure 2), and washed by D-Hank’s solution containing penicillin-streptomycin double antibiotics. The study protocol was approved by the ethics committee of Zhongshan Hospital (No: 2009-173).

In primary culture, JE tissues were digested with 2 ml of DispaseII working solution at 4°C for 16-18 hours. The epithelium was separated from the lamina propria by forceps, and then cut into pieces, digested with 4 ml of 0.025% trypsin-0.01% EDTA for 5-8 minutes with stirring. The digestion was terminated by adding D-Hank’s solution containing 10% FBS, followed by filtration using 180 μm stainless steel sieve and then centrifugation. The precipitates were mixed in (defined keratinocyte growth medium) DKGM to form cell suspension. Cells were seeded in 24-well plates at 2 × 10^5^ cells/ml, and placed in a 37°C, 5% CO_2_ incubator. The medium was refreshed after 3 days for the first time, then once a day. In passage culture, cells were passaged at 60-70% confluence by adding 0.25% trypsin-0.02% EDTA at 37°C for 5-8 min. When the cells appeared rounded under a microscope, the digestion was terminated. Then cells were suspended and centrifuged. DKGM was added to form cell suspension, and dispensed into new petri dishes. On the other hand, OGE cells were treated as JE cells above. Differently, the OGE tissue was cut into small pieces of 5 × 5 mm^2^. The OGE cell suspension was seeded at densities ranging from 5-10 × 10^5^ cells/ml in petri dishes of 60 mm in diameter. The cultured cells were observed daily using inverted phase contrast microscope to track their morphology and growth conditions. In addition, the passaged single cell suspension was inoculated at densities ranging from 2-5 × 10^5^ cells/ml onto cover slips in sterile petri-dishes. Then treated with H&E staining when cells were grown to 60% confluence, and observed using a light microscope to track changes on the morphology and structure.

### Cell growth curve in JE and OGE cells

Primary JE, OGE cells at 100% confluence were digested to form single cell suspension and seeded at a density of 2 × 10^4^/cm^2^ in 24-well plates. Cells in two random wells were counted daily using a hematocytometer. A cell growth curve was plotted by the average cell number each day versus the number of days. The doubling time was obtained from the growth curve which could indicate the length of time required for cells to double in number during the logarithmic phase.

### Immunohistochemistry analysis of human gingival epithelium tissues and vitro cultured cells

Immunohistochemical peroxidase-conjugated streptavidin (SP) method was performed on human gingival tissue by incubation with Anti-CK 5/6 (clone D5/16B4), anti-CK 7 (OV-TL12/30), anti-CK 8/18 (clone Zym5.2), anti-CK 10/13 (clone DE-K13), anti-CK 16 (clone LL025), anti-CK 17 (clone E3), anti-CK 19 (clone A53/BA2) and anti-CK 20 (clone Ks20.8). These anti-human cytokeratin monoclonal antibodies were obtained from Zymed (U.S.A). Human parotid gland tissue was stained as positive control [25] and the primary antibody replaced by PBS was used as negative control. The immunohistochemistry staining procedure was performed by Ab manufacturer’s instructions. Simply, dewaxed sections were incubated with pepsin solution at 37°C for 5-10 min, and incubated with blocking serum at room temperature for 30 min. Primary antibody at 1:50 dilution was added in the study group and the positive control. PBS instead of the primary antibody was added in the negative control. After being incubated at 4°C overnight, the sections were incubated in the working solution with biotin labeled secondary antibody at room temperature for 30 min, and followed with horseradish peroxidase labeled streptavidin solution at room temperature. DAB chromogenic solution was added for 5-10 min. Sections were rinsed with running water, re-stained with hematoxylin and mounted. On the other hand, passaged cells adhered to cover slips were fixed using 10% neutral formalin. The cells were treated as JE tissues above. In these cells, CK-Pan, CK19, CK10/13 and CK16 were detected. The cytoplasm of CK positive cells was stained and was classified as negative (-) with no coloring, weak positive (+) with coloring of light yellow, moderate positive (++) with coloring of yellow or strong positive (+ + +) with coloring of brown [26].

### Co-culture of human JE cells and root slices

Teeth slices were from the same samples recruited for the JE primary cells. Samples were imbricated scrap using a Grace curette to remove the periodontal membrane. They were fixed in 10% neutral formalin for 12 h, and decalcified using Plank-Rychlo decalcification solution for 2 weeks. The dental crowns were removed and the teeth were sectioned along the root surface into dental films of 5 × 5 mm^2^ in size and 1-1.5 mm in thickness. The films were washed for 2-3d and soaked in D-Hank’s solution containing penicillin-streptomycin double antibiotics at 4°C before use. The passaged human JE cells suspension was inoculated at a density of 5 × 10^5^ cells/ml on the root slices with the cementum surface up in 24-well plates, 2 to 3 slices per well, and placed for 14 d in a 37°C 5% CO_2_ saturation humidity incubator. The medium was refreshed 3 d later, and then once a day.

Observation using TEM: Root slices were collected 3, 5, 7, 9, 11, and 14 d after inoculation respectively and fixed in 2% glutaraldehyde at 4°C for 2 h. Root slices, with the cementum surface up, were cut into small pieces of 5 × 1 × 1 mm, post-fixed in 1% osmic acid at 4°C for 2 h, dehydrated by ethanol, soaked by propylene oxide at RT for 24 h, and embedded in araldite. The embedded specimens were cut into ultrathin slices, which were stained using lead citrate, followed by observation using TEM (PHILIP CM-120, Holland) to track the formation of JE cells attachment to the cementum surface.

## Results

### Morphological analysis of human gingival epithelium tissues

**Figure 3:**
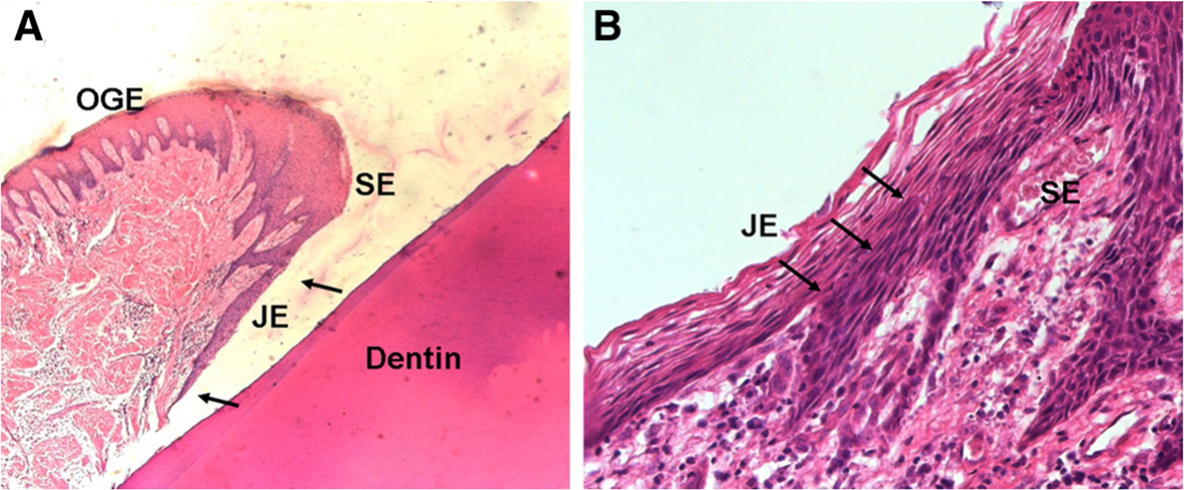
**HE staining of human gingival tissue. A**. The region between the arrows is JE (x150); **B**. The boundary between JE and SE, the arrow points to the boundary (x1200).

Under the microscope, JE was short and strip-like, gradually thickened from the cemento-enamel junction to the coronal. After stained with HE, no keratinization or epithelial ridges existed in JE tissue which was divided into basal layer and suprabasal layer, while keratinized or partially keratinized epithelium, dense and irregular epithelial ridges projecting into adjacent connective tissue were found in dark-stained SE and OGE tissues (Figure 3A). Moreover, JE cells were different from SE and OGE in morphology and had clear boundary with SE (Figure 3B). The cells in JE tissue were uniform in shape, either flat or spindle, aligned parallel to the tooth surface and the cellular junctions were loose with obvious intercellular space. However, SE and OGE cells were all irregular polygons and tightly aligned with less or even no intercellular space. Besides, JE cells were abundant in organelles and the nucleus was large, and similarly, the nuclei of SE and OGE cells were also large but hyperchromatic. Further, SE and OGE cells presented typical structural features of squamous cells and could reciprocally transform with no clear boundary.

According to measurements in the image analysis, JE tissue was 1.021 ± 0.128 mm in width, 0.066 ± 0.009 mm in thickness, and 0.042 ± 0.002 mm^2^ in area, while, the SE was 0.532 ± 0.176 mm in width (Table 1).

### Morphological analysis of cultured human gingival JE and OGE cells in vitro

**Figure 4:**
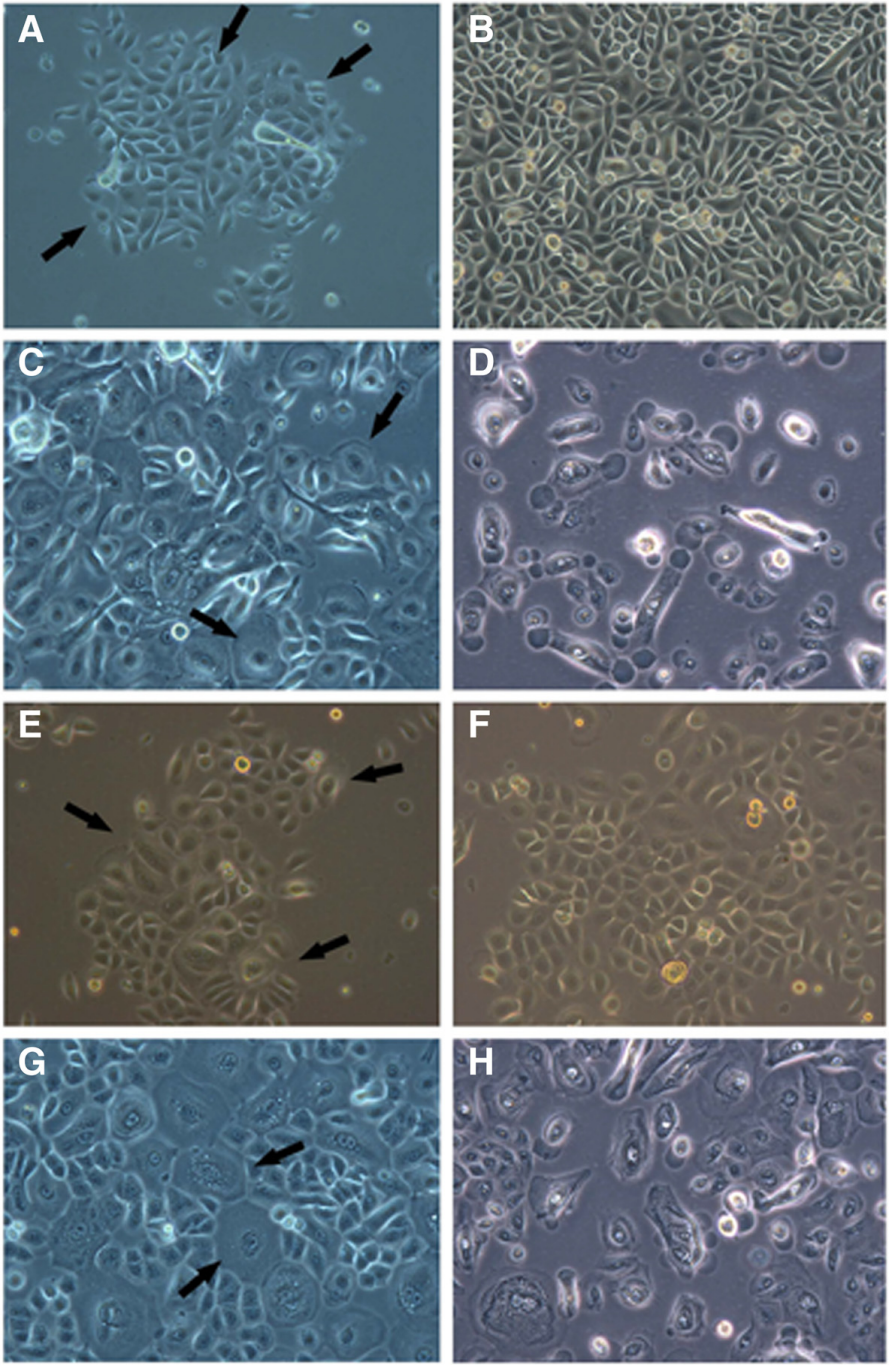
**Observation of JE and OGE cell morphology (primary cells). A**. JE cell clones formed (the arrow × 200); **B**. At 10—12 d, JE cells presented non-uniform morphology and scattered arrangement (× 200); **C**. The 3rd passage JE cells multiple ‘giant cells’ appeared (the arrow × 200); **D**. The 5th passage JE cells showed signs of aging and death (× 400); **E**. the OGE cell clones formed and expanded (the arrow × 200); **F**. At 7—9 d, the OGE cells presented uniform morphology, tight arrangement, and ‘paving stone-like’ keratinizing (× 200); **G**. In the 3rd passage OGE cells ‘giant cells’ appeared (× 200); **H**. The 7th passage OGE cells showed signs of aging and death (× 400).

In order to observe the JE cells clearly and identify the characteristics, JE and OGE cells were cultured in vitro. As a result, initially seeded primary JE cells presented diverse morphology, such as polygonal flat, spindle-shaped, oval and spherical. After 24-96 h incubation, the cells adhered to the petri dish bottom, the cytoplasm turned dark, the membrane was rough, and 2-3 fold cells were fully stretched. The nuclei were large and commonly 2 to 3 nucleoli in each cell. After 7 d, cells gradually came into rapid growth period and extended along the petri dish edge to the center. Scattered mitosis and cell clones with angular or fusiform morphology appeared as shown in Figure 4A. After 10-12 d, cell clones with diverse morphology, non-uniform size formed confluent patches (Figure 4B). In the first passage, cells adhered to the dish bottom and stretched in 24-48 h after inoculation, then the cells presented similar morphology with primary cells; In the second passage, JE cells showed irregular morphology like ‘giant cells’ pseudopodia, and cytoplasmic vacuolation (Figure 4C); In the following passages, the morphology was more irregular and cell proliferation seemed to slow down, even presenting aging and death signs (Figure 4D).

As the OGE cells, initially seeded primary cells presented polygonal or spherical shapes and adhered to the dish bottom within 24-72 h; After 5 d, cell clones formed with triangular or polygonal morphology, however, these cells were more uniform than the JE cell clones of the same period (Figure 4E); After 7-9 d, the OGE clones enlarged and converged, then tightly arranged and showed typical ‘paving stone-like’ keratinization (Figure 4F); After 9-11 d, the cells were about 100% confluent; In the second passage, cells adhered and stretched in 48 h. And then the ‘giant cells’ appeared (Figure 4G); After being passaged for 4 times, cells were mainly ‘giant cells’ and proliferation slowed down also with aging and death signs (Figure 4H).

**Figure 5:**
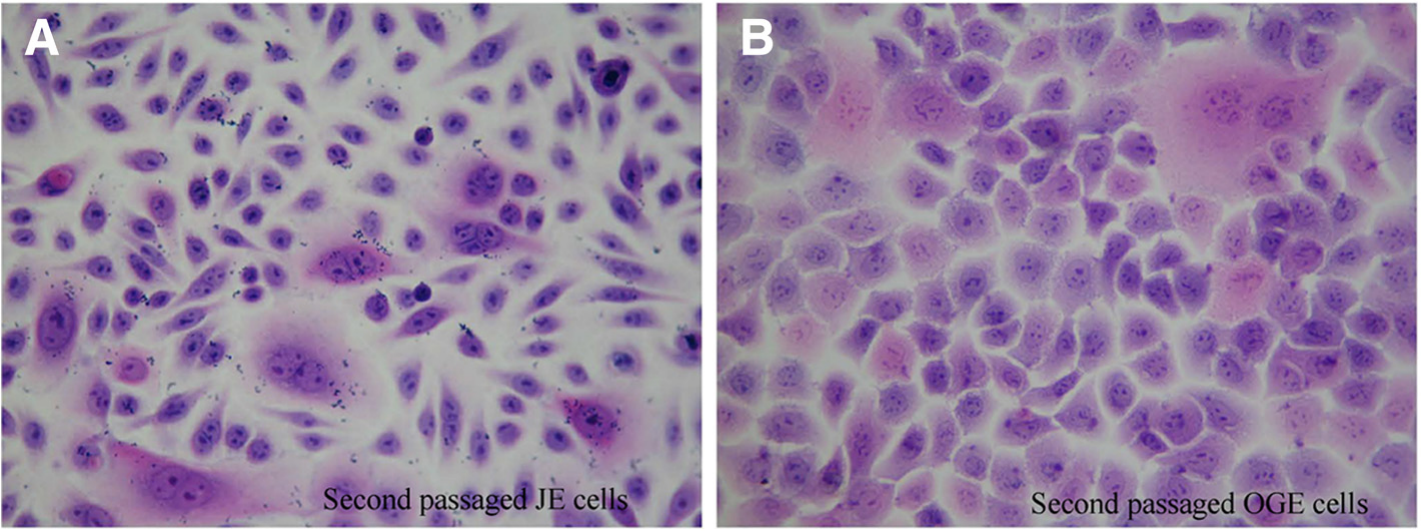
**H&E staining of JE and OGE cells (second passaged cells, H&E × 400). A**. JE cells present non-uniform in size and morphology, scattered arrangement, large and deeply-stained nuclei and multiple nuclear divisions; **B**. OGE cells were uniform in size and shape, tightly arranged, and ‘paving stone-like’ keratinizing.

After that, JE and OGE cells were stained with HE and observed under the microscope. As a result, JE cells appeared diverse morphology (spindle-shaped, triangle, oval), non-uniform size, relaxed arrangement, large and dark stained nuclei and multiple nuclear divisions as mentioned above (Figure 5A). However, OGE cells were uniform in size, tightly arranged, typically keratinized, and with round nuclei in the center, as well as visible nuclear divisions (Figure 5B). Therefore, JE were significantly different from OGE in cell morphology.

### Growth condition of cultured JE and OGE cells in vitro

**Figure 6:**
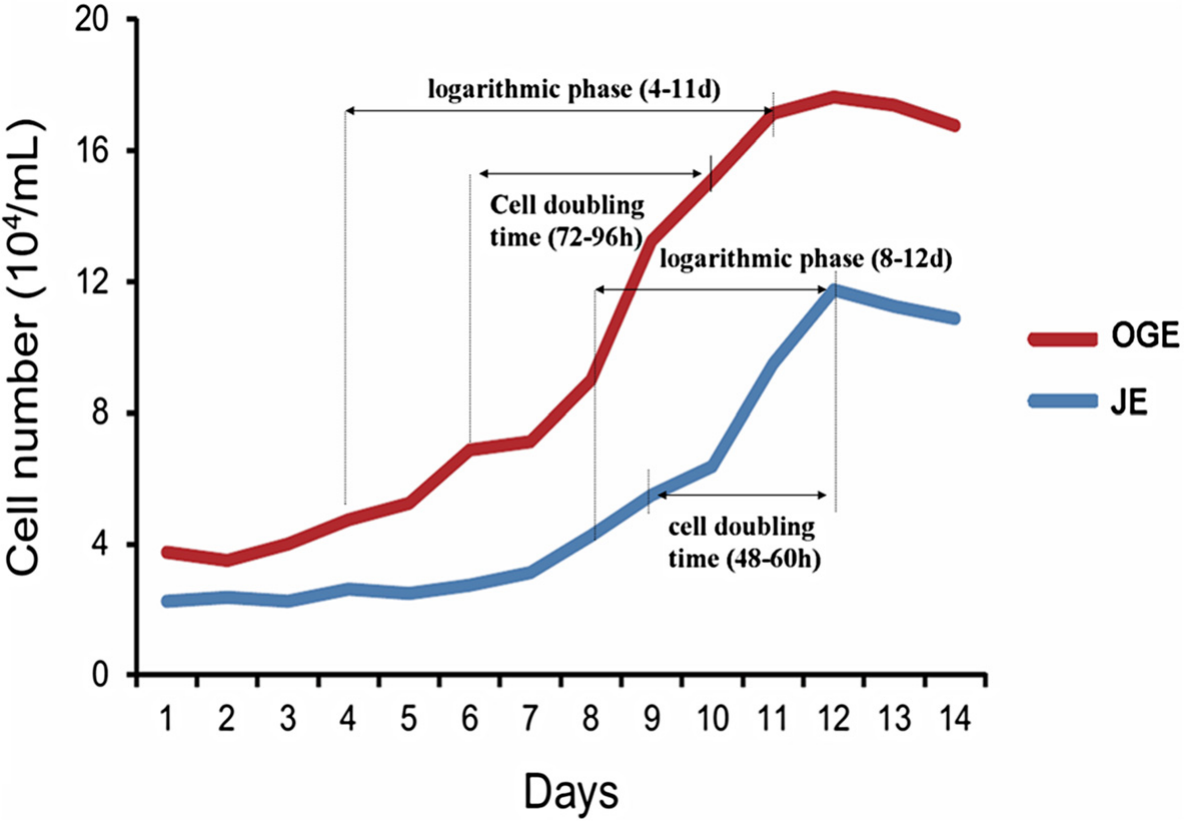
**Growth curve of JE and OGE cells.** The JE cells were in latent phase (1-7 d after inoculation), exponential phase (8-12 d), and plateau phase (12 d thereafter). OGE cells were in latent phase (1-3 d after inoculation), exponential phase (4-11 d), and plateau phase (12 d thereafter).

**Figure 7:**
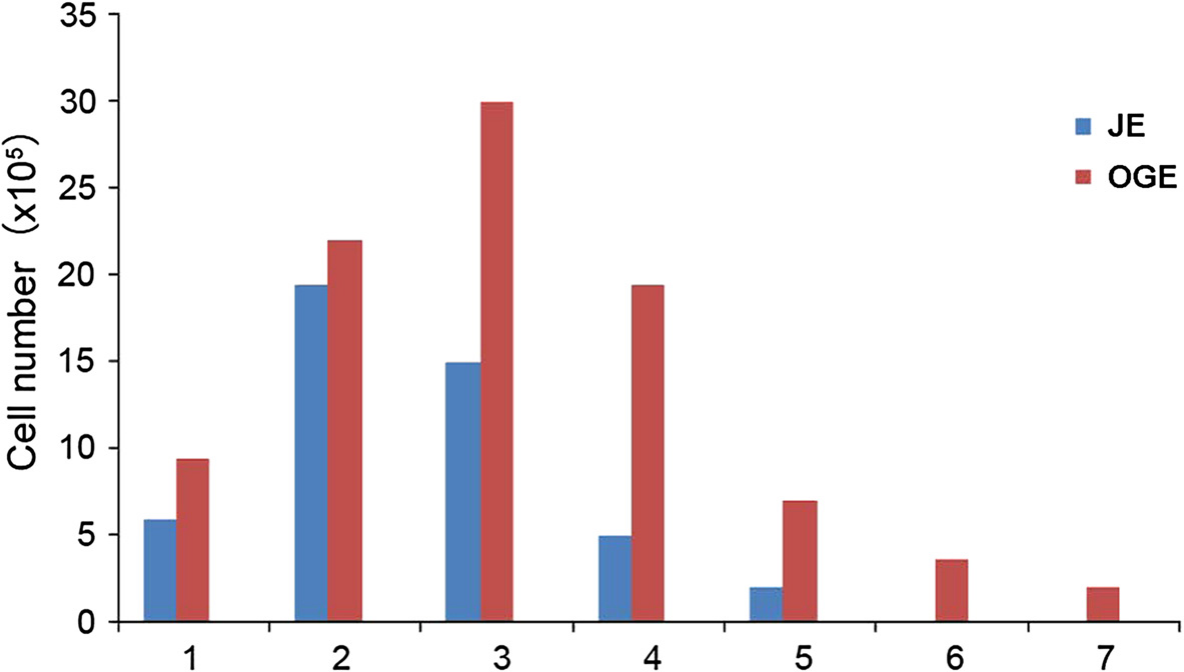
**Passage numbers of cells and number of cells per passage for JE and OGE cells.** (JE cells were passaged five times. OGE cells were passaged 7 times).

We then analyze the growth conditions of these two cells. There were more OGE cells cultured compared with JE. As a result, the incubation period for JE cells to attach and proliferate was 1-7 d in vitro, while for OGE cells was 1-3 d, a little shorter. The logarithmic phase and growth peak of JE cells appeared in the 8^th^ - 12^th^ d (only last for 5 days), while of OGE was 4^th^ - 11^th^ d (8 days). After 12 d, JE and OGE cells were both entered into a period of stagnation. Besides, the number of JE cells has grown from 6×10^4^/ml to 12×10^4^/ml during 9-12 days, and OGE cells has grown from 7×10^4^/ml to 14×10^4^/ml during 6-10 days. According to statistics, the cell doubling time of JE cells was 48-60 h, while OGE was 72-96 h. Overall, OGE exhibit more gently curves than JE (Figure 6). As shown in Figure 7, JE cells were successfully passaged for 5 times while OGE 7 times in the present experiment. The quality of passaged JE cells drastically declined after the 3rd passage, but after 4^th^ passage in OGE cells.

### Immunohistochemistry analysis of human gingival epithelium tissues and cultured cells in vitro

**Figure 8:**
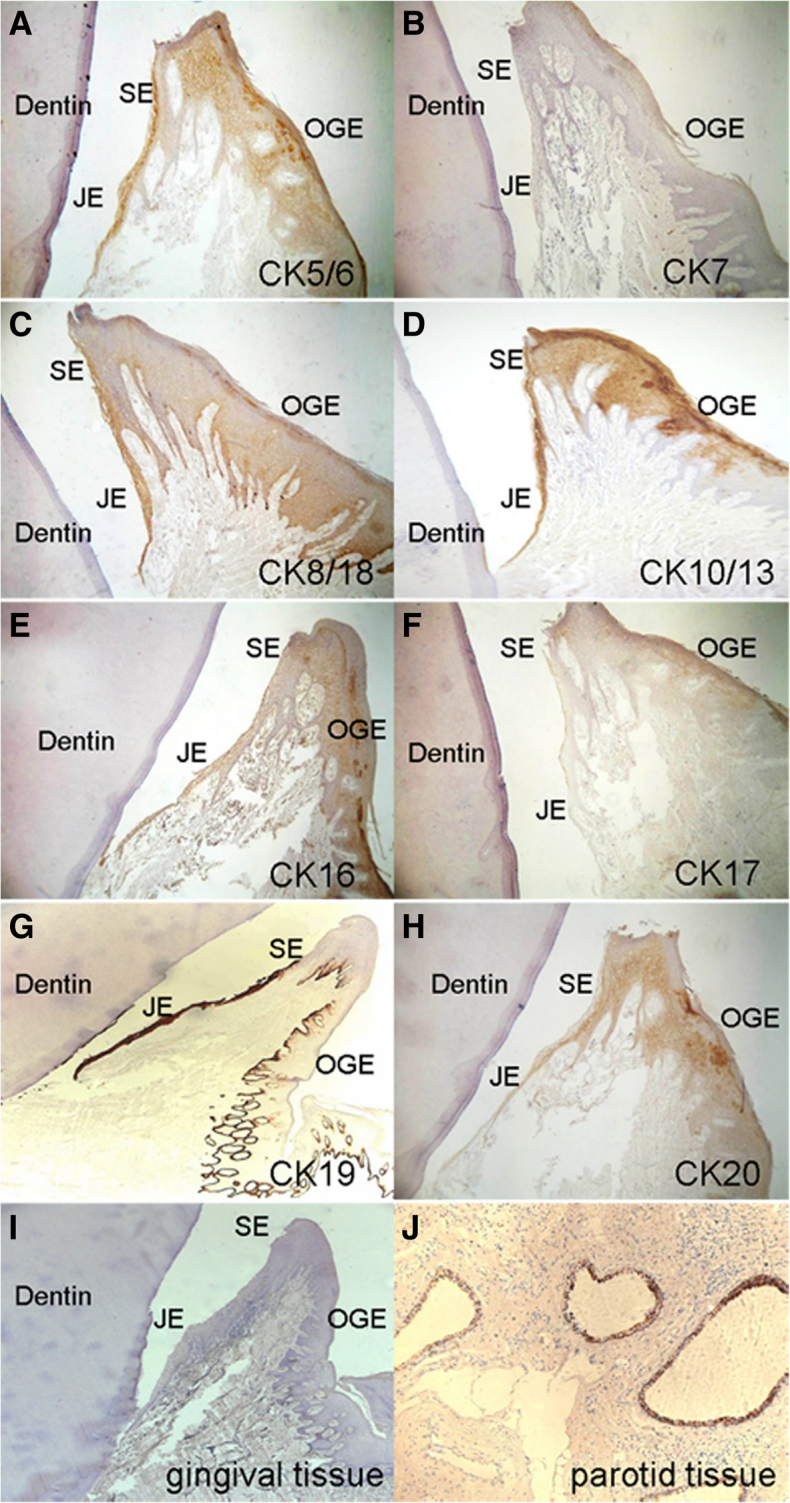
**Immunohistochemistry stain of human gingival tissues (×100)**. Staining against different cytokeratins was performed. **A**. CK5/6; **B**. CK7; **C**. CK8/18; **D**. CK10/13; **E**. CK16; **F**. CK17; **G**. CK19; **H**. CK20; **I**. Human gingival tissue was used as negative control; **J**. Human parotid tissue was used as positive control.

In order to deeply identify the functional characteristics of JE, several CKs were analyzed. In human gingival epithelium tissues, the expression of CK5/6 and CK20 was similar although CK5/6 was stronger stained. They were positive stained in the suprabasal layer (especially near the surface) and negative stained in the basal layer; The expression of CK7 and CK17 was negative or only weak positive in very few cells; In CK10/13 and CK16, they were expressed in all layers of JE but only in the suprabasal layer of OGE and SE; The expression of CK10/13 was strong positive and CK16 was weak positive or positive; CK19 was detected in all layers of JE with strong positive expression, while its expression in OGE and SE was limited to the suprabasal layer and no staining was seen in the basal layer. The boundary between JE and SE was clearly due to the difference in CK19 staining; The expression pattern of CK8/18 was at a lower level which was similar to CK19 except the basal layer of OGE and SE. Besides, it also showed weak positive expression in the suprabasal layer (close to the basal layer) in some slices. The detailed descriptions were in Table 2 and Figure 8.

**Table 2:** Expression pattern of different cytokeratins in JE, OGE, and SE

**Antibody**	**CK**	**Sample no. (n)**	**Slice no. (n)**	**JE**		**OGE**		**SE**	
				**b**	**sb**	**b**	**sb**	**b**	**sb**
D5/16B4	5/6	6	20	-	++	-	+	-	+
OV-TL12/30	7	6	10	-	-	-	-	-	-
Zym5.2	8/18	6	15	++	++	+	-^*^	+	-^*^
DE-K13	10/13	6	20	+++	+++	-	+++	-	+++
LL025	16	6	18	+	+	-	++	-	+
E3	17	6	11	-	-	-	-	-	-
A53/BA2	19	6	10	+++	+++	+++	-	+++	-
Ks20.8	20	6	14	-	+	-	+	-	+

Note: b: basal layer, sb: suprabasal layer, -: negative, +: weak positive, ++: moderate positive, +++: strong positive, *: weak positive expression in the suprabasal layer in some slices.

**Figure 9:**
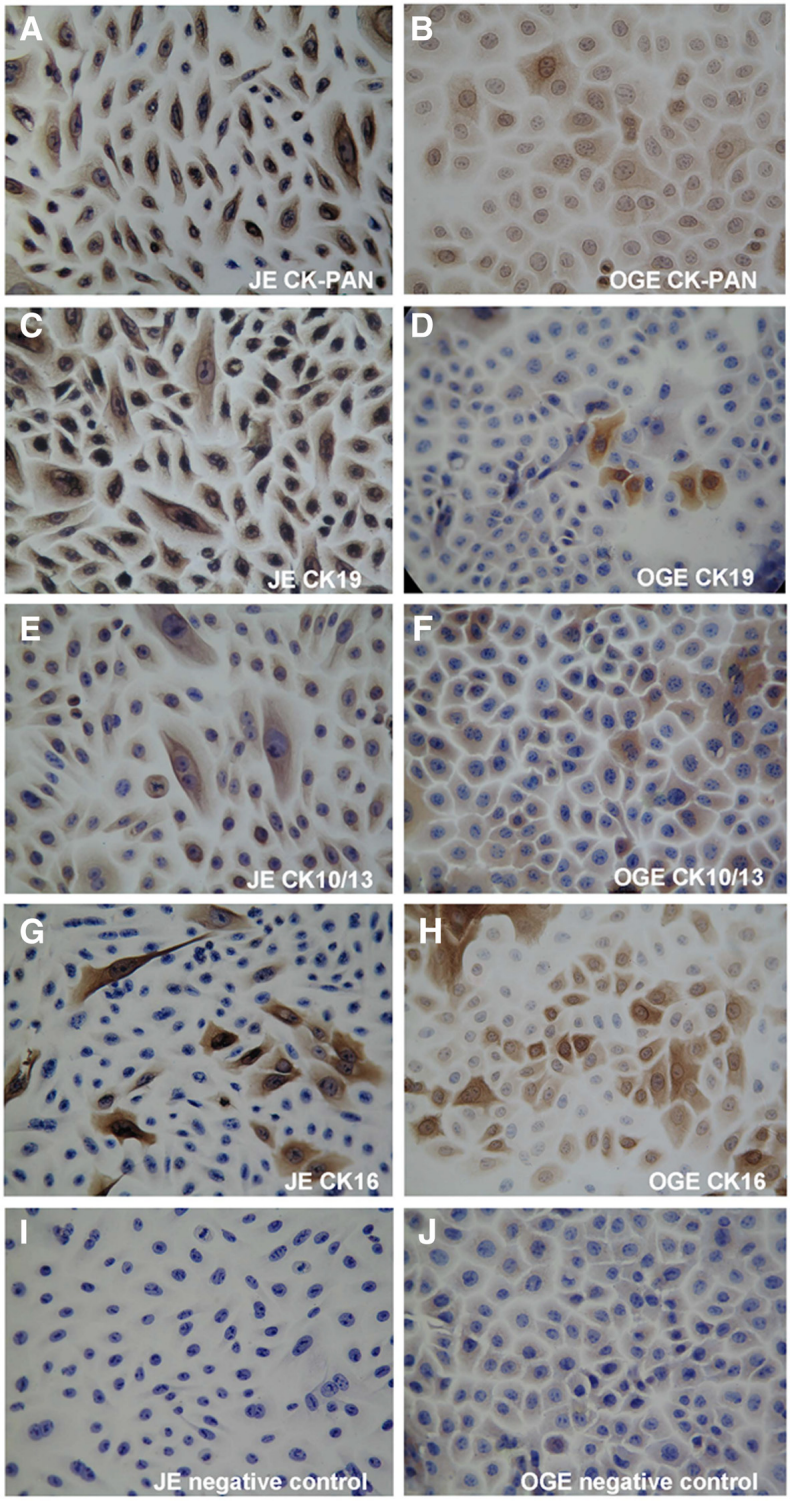
**Immunohistochemical staining of JE and OGE cells (second passage cells, SP × 400). A**. JE cells, CK-Pan positive; **B**. OGE cells, CK-Pan positive; **C**. JE cells, CK19 strongly positive; **D**. OGE cells, CK19 in a very small number of scattered positive cells; **E**. JE cells, CK10/13 weak positive to positive; **F**. OGE cells, CK10/13 weak positive to positive; **G**. JE cells, CK16 scattered positive; **H**. OGE cells, CK16 scattered positive; **I**. JE cells, negative control; **J**. OGE cells, negative control.

In cultured cells, both JE and OGE cells were stained positively for CK-Pan (Figure 9A, B). Strongly positive staining of CK19 was seen in JE cells (Figure 9C), but only a small number of scattered OGE cells were stained positive (Figure 9D); The CK10/13 stain of both JE and OGE cells were weak positive or positive (Figure 9E, F); Besides, the two kinds cells were scattered positively stained with CK16 (Figure 9G, H). The negative controls were not stained in all conditions (Figure 9I, J).

### TEM observation of the formation of JE cells attachment to root slices

**Figure 10:**
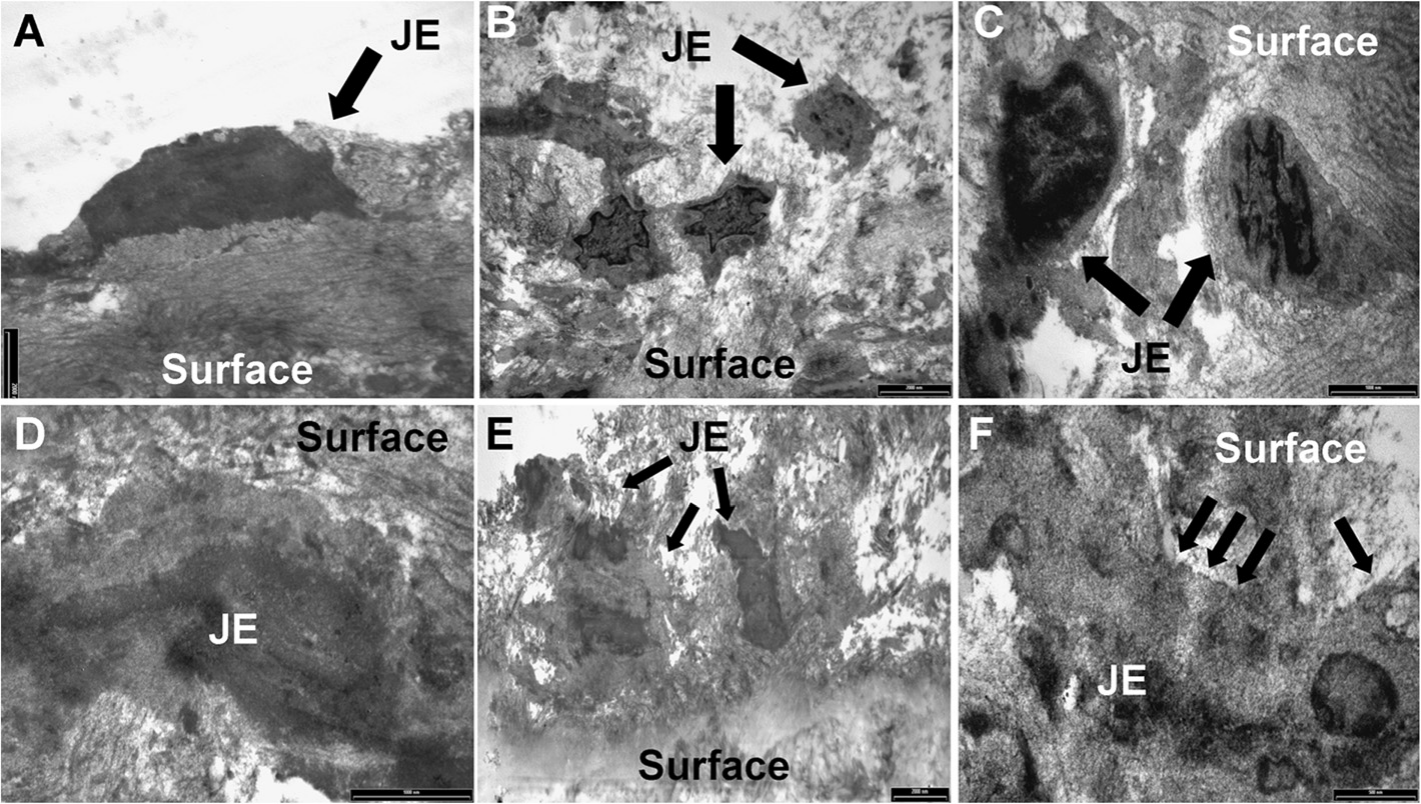
**TEM observations of the structural formation of JE cells attachment to the cementum surface. A**. At 3 d, there was a small number of cells on cementum surface, cells were spherical, did not stretch (TEM × 13500); **B**. At 5 d, cells on cementum surface increased in number, and a portion of cells stretched (TEM × 9700); **C**. At 7 d, cells fully stretched to be flat-shaped, and attached to the cementum surface, but did not form clear basement membrane-like and hemidesmosome-like structures (TEM × 24500); **D**. At 9 d, JE cells appeared to have a small number of electron-dense deposits like hemidesmosome at the local cell membrane attached to cementum surface (arrows, TEM × 33000); **E**. At 11—14 d, there was a significantly increase in cell number on cementum surface, and multi-layer cells appeared (TEM × 7400). **F**. a large number of electron-dense deposits (arrows) appeared at JE cell membrane—cementum surface junction, forming the basement membrane-like and hemidesmosome-like structures (TEM × 46000).

Finally, the attachment formation between JE cells and tooth surface was studied by a co-culture model. JE cells and root slices were co-cultured in vitro. Then JE cells on human cementum surface were observed. Three days after inoculation, the cells were spherical and not fully stretched (Figure 10A); Five days later, the number of JE cells on cementum surface increased and a portion of cells were stretched (Figure 10B); At 7 d, JE cells were fully stretched to be flat-shaped, attached to the cementum surface with cell membrane, but did not appear clear basement membrane and hemidesmosome-like structures (Figure 10C); At 9 d, JE cells appeared to have a small number of electron-dense deposits like hemidesmosome at the local cell membrane attached to cementum surface (arrows, Figure 10D); At 11-14 d, there was a significantly increased cell number in root slices, and multi-layer cells appeared (Figure 10E). In addition, a large number of electron-dense deposits appeared at cell membrane-cementum surface junction. Finally, basement membrane-like and hemidesmosome-like structures were formed (arrows, Figure 10F).

## Discussion

### JE is a unique human gingival epithelium tissue

According to the observation of tissues, we found that normal human JE tissue belonged to simple stratified epithelium. The cells were uniform in shape, relatively lower in differentiation, without keratinization and epithelial ridges in vivo. This is probably due to its location at the bottom of the gingival sulcus, tooth surface attachment and rarely subjected to external stimulation. However, the SE and OGE are exposed to oral cavity environment and exhibit typical characteristics of squamous epithelium cells. They were polygon in shape, tightly aligned, keratinized in the surface layer with dense epithelial ridges which projected into the connective tissue, and present a clear boundary with JE. In preliminary experiments, epithelial ridges were also seen in JE of gingival atrophy or periodontal pockets. These suggest that external stimulation and inflammation may result in the formation of epithelial ridges. Additionally, image analysis results showed that JE was only about 1 mm in length, 60 μm in width, and 15 to 20 layers of cells deep. This result showed that the volume of JE tissue was extremely small and difficult to collect, which is one of the major reasons why JE is difficult to study. On the other hand, JE and OGE cells were isolated and cultured in vitro. As a result, the cell morphology of JE cells was significantly different from typical-keratinizing OGE cells. JE cells were similar to connective tissue fibroblasts in cell morphology and varied in morphology. This indicates that JE is a unique poorly-differentiated epithelium in the gingival.

### Growth conditions of JE and OGE cells in vitro

On the growth curve, JE cells had a longer incubation period than OGE cells, account for half of the growth cycle. Then JE cells accelerated proliferate to the peak, and immediately followed by recession. By comparison, OGE cells entered into a longer period of proliferation after a short incubation period, then followed by slow recession. In addition, the cell doubling time of JE cells was shorter than OGE. However, JE could passage fewer times than OGE cells. Possible reasons for these differences may be explained as follows. In vivo, JE is located at the gingival sulcus bottom. This is a closed environment where the cells are rarely differentiated. Thus, there will be a long incubation period for JE cells to adapt the new environment. After adaption, JE cells have a unique ability to proliferate rapidly and reach contact inhibition in a relatively short period. By comparison, OGE cells are always contact with the outside, the cell differentiation is high, and the ability to adapt to the environment is strong. Thus, the growth curve was gentle changed in OGE cells.

### Analysis of variety expressed CKs

Varieties of CKs were expressed in the oral epithelial cells at different levels depending on the location within the oral cavity. In this study, expression of CK5/6 [22] was negative in the basal layer of all three types of gingival epithelium. The positive stain in the suprabasal layer may derive from CK6 [20]. As a marker for single layer epithelium, CK8 and 18 are generally not expressed in squamous epithelium. Bampton et al. showed that CK8/18 was not expressed in JE, but expressed in vitro cultured gingival epithelial cells [27]. Mackenzie et al. showed that CK8/18 could express in OGE and SE but not constantly [20], while Pritlove-Carson et al. showed that the expression of CK8/18 in JE increased in inflammation [3]. In this study, we found that CK8/18 expressed in all layers of JE but only in the basal layer of OGE and SE (in some slices, it also showed weak positive expression in the suprabasal layer). The result is same with the studies by Mackenzie et al. The expression pattern of CK19 was similar to CK8/18, but CK19 expressed higher and the boundary between JE and SE was clearly due to significant differences in staining. Previous studies have shown that CK19 is highly expressed in newly erupted JE [22], regenerated JE after surgical operation [28], epithelium inside the periodontal pocket [20], inflammatory gingival epithelium [21] and vitro cultured epithelial cells of the periodontal pocket [15]. It can be used as a marker for gingival epithelium with continuous differentiation [8]. The expression patterns of CK8/18 and 19 certificate that JE is a specialized epithelium different from general squamous epithelium. Silimilarly, the strong positive expression of the epithelial differentiation-associated marker CK19 was also found in cultured JE cells which further indicates that JE cells are in a continuous state of differentiation.

However, some CKs were differently expressed in vitro and vivo. CK16 was expressed in all layers of JE but mainly in the suprabasal layer of OGE [3,29]. However, it was scattered positively stained in both OGE and JE. Strong positive expression of CK10/13 [15,22,29] was found in suprabasal layer and negative in basal layer of OGE. While both JE and OGE cells were weak positive or positive stained. These differences may be explained by the non-specific of antibody or the different growth conditions. Therefore, they could not be used for a clear distinction between JE and OGE.

The same expression pattern of a variety of CKs in OGE and SE indicates they are the same type of epithelium. However, OGE and SE were greatly different with JE. Most CKs were widely expressed in JE (such as CK10/13, 16, 19) and highly expressed (such as CK5/6, 8/18, 19). Usually, tissues or cells with a low differentiation level are more active in proliferation. It explains why JE is rapidly regenerated. Moreover, the expression of CKs was more widespread and in a higher level in the suprabasal layer (especially close to the surface) than that of the basal layer, such as CK5/6, 8/18, 19, and 20 [30,31]. Most of these highly expressed CKs reflect both a high proliferation ability and high level of differentiation [8,20,22,30,31]. As a consequence, the suprabasal layer of JE has a lower differentiation but higher regeneration ability than the basal layer. This appears to go against the biological nature of regular epithelium, but further illustrates the unique biological characteristics of JE. However, the indicative function of these CKs was objective. Further researches on JE were still needed.

### Co-cultured JE cells and root slices

Human gingival tissue blocks (1 × 1 × 2 mm^3^) and dentin slices or a millipore filter were co-cultured previously [32]. As a result, the dentin slices and epithelial cells formed hemidesmosomes-like and basement membrane-like structures. The structures were similar to JE-tooth surface adhesion. But there was no such structure formed between the millipore filter and the cells. Oksanen et al. [16] also observed a large number of electron-dense plaques at the junction of cultured rat oral epithelial cells and tooth slices, where formed hemidesmosome-like structures. JE is usually attached to the enamel of the tooth neck. But when periodontal tissues are destroyed and periodontal pockets are formed. JE are receded to the root and formed attachments on the cementum surface of the root. Therefore, in this study we selected human root slices instead of dentin slices. As a result, many cells adhered onto the cementum surface after 11 d co-cultured. While, JE cells cultured in petri dish were reached almost 100% confluence in the same period (11 d). Then we suspect the attachment may be associated with the surface treatment of the carrier. The petri dish surface is smooth and easy for cells to attach and stretch. In contrast, the cementum surface is rough and not conducive for cells to attach. It verifies the importance of root surface smoothness through periodontal scaling to facilitate the regeneration of cell new attachment. In composite culture, multi-layer cells and intensive hemidesmosome- like structures appeared within 11-14 d. This indicates the attachment between JE cells and tooth surface was formed in about 2 weeks. However, the environment in vivo is complicated which will be affected by many factors. For example, infected root surface and subgingival may delay the formation of periodontal new attachment in clinic. Therefore, clinical studies on JE attachment are still need to be studied.

## Conclusions

JE is a special stratified epithelium with low differentiation and high regeneration ability in the gingival tissue. In co-culture model, human JE cells can form basement membrane-like and hemidesmosome-like structures in about 2 weeks.

## Competing interests

The authors declare that they have no competing interests.

## Authors' contributions

QJ carried out the molecular genetic studies, cell culture, the analysis in vitro and drafted the manuscript. YY and HR contributed the histologic-morphometric part, YL and XG contributed the TEM investigation. YL participated in the design of the study and performed the statistical analysis. XG conceived of the study, and participated in its design and coordination. All authors read and approved the final manuscript.

## Pre-publication history

The pre-publication history for this paper can be accessed here:

http://www.biomedcentral.com/1472-6831/14/30/prepub

## References

[B1] Jiang Q Li D Comparative study on the histomorphology of the JE of human and several laboratory animals] Shanghai Kou qiang Yi Xue = Shanghai J Stomatology 2004 13 6 539 15619701

[B2] Willberg J Syrjänen S Hormia M Junctional epithelium in rats is characterized by slow cell proliferation J Periodontol 2006 77 5 840 846 10.1902/jop.2006.050213 16671877

[B3] Pritlove-Carson S Charlesworth S Morgan PR Palmer RM Cytokeratin phenotypes at the dento-gingival junction in relative health and inflammation, in smokers and nonsmokers Oral Dis 1997 3 1 19 24 9456642 10.1111/j.1601-0825.1997.tb00004.x

[B4] Newman MG Takei H Klokkevold PR Carranza FA Carranza's Clinical Periodontology 2011 Philadelphia: Elsevier Health Sciences

[B5] Hormia M Owaribe K Virtanen I The dento-epithelial junction: cell adhesion by type I hemidesmosomes in the absence of a true basal lamina J Periodontol 2001 72 6 788 797 10.1902/jop.2001.72.6.788 11453242

[B6] Schroeder HE Listgarten MA The junctional epithelium: from strength to defense J Dent Res 2003 82 3 158 161 10.1177/154405910308200302 12598541

[B7] Gursoy UK Könönen E Luukkonen N Uitto V-J Human neutrophil defensins and their effect on epithelial cells J Periodontol 2013 84 1 126 133 10.1902/jop.2012.120017 22443519

[B8] Shimono M Ishikawa T Enokiya Y Muramatsu T Matsuzaka K-i Inoue T Abiko Y Yamaza T Kido MA Tanaka T Biological characteristics of the junctional epithelium J Electron Microsc 2003 52 6 627 639 10.1093/jmicro/52.6.627 14756251

[B9] Heymann R Wroblewski J Terling C Midtvedt T Öbrink B The characteristic cellular organization and CEACAM1 expression in the junctional epithelium of rats and mice are genetically programmed and not influenced by the bacterial microflora J Periodontol 2001 72 4 454 460 10.1902/jop.2001.72.4.454 11338297

[B10] Oksanen J Sorokin L Virtanen I Hormia M The junctional epithelium around murine teeth differs from gingival epithelium in its basement membrane composition J Dent Res 2001 80 12 2093 2097 10.1177/00220345010800121401 11808769

[B11] Marchetti C Farina A Cornaglia AI Microscopic, immunocytochemical, and ultrastructural properties of peri-implant mucosa in humans J Periodontol 2002 73 5 555 563 10.1902/jop.2002.73.5.555 12027260

[B12] Ishikawa H Hashimoto S Tanno M Ishikawa T Tanaka T Shimono M Cytoskeleton and surface structures of cells directly attached to the tooth in the rat junctional epithelium J Periodontal Res 2005 40 4 354 363 10.1111/j.1600-0765.2005.00815.x 15966914

[B13] Pan YM Firth J Salonen J Uitto VJ Multilayer culture of periodontal ligament epithelial cells: a model for junctional epithelium J Periodontal Res 1995 30 2 97 107 10.1111/j.1600-0765.1995.tb01258.x 7539838

[B14] Tomakidi P Fusenig N Kohl A Komposch G Histomorphological and biochemical differentiation capacity in organotypic co‒cultures of primary gingival cells J Periodontal Res 1997 32 4 388 400 10.1111/j.1600-0765.1997.tb00549.x 9210093

[B15] Papaioannou W Cassiman J-J Oord JV Vos RD Steenberghe D Quirynen M Multi-layered periodontal pocket epithelium reconstituted in vitro: histology and cytokeratin profiles J Periodontol 1999 70 6 668 678 10.1902/jop.1999.70.6.668 10397522

[B16] Oksanen J Hormia M An organotypic in vitro model that mimics the dento-epithelial junction J Periodontol 2002 73 1 86 93 10.1902/jop.2002.73.1.86 11846204

[B17] Pitaru S McCulloch CA Narayanan SA Cellular origins and differentiation control mechanisms during periodontal development and wound healing J Periodontal Res 1994 29 2 81 94 10.1111/j.1600-0765.1994.tb01095.x 8158503

[B18] Moll R Divo M Langbein L The human keratins: biology and pathology Histochem Cell Biol 2008 129 6 705 733 10.1007/s00418-008-0435-6 18461349 PMC2386534

[B19] Mackenzie I Rittman G Gao Z Leigh I Lane E Patterns of cytokeratin expression in human gingival epithelia J Periodontal Res 1991 26 6 468 478 10.1111/j.1600-0765.1991.tb01797.x 1722249

[B20] Mackenzie I Gao Z Patterns of cytokeratin expression in the epithelia of inflamed human gingiva and periodontal pockets J Periodontal Res 1993 28 1 49 59 10.1111/j.1600-0765.1993.tb01050.x 7678864

[B21] Nagarakanti S Ramya S Babu P Arun K Sudarsan S Differential expression of E-Cadherin and cytokeratin 19 and net proliferative rate of gingival keratinocytes in oral epithelium in periodontal health and disease J Periodontol 2007 78 11 2197 2202 10.1902/jop.2007.070070 17970688

[B22] Feghali-Assaly M Sawaf M Serres G Forest N Ouhayoun J Cytokeratin profile of the junctional epithelium in partially erupted teeth J Periodontal Res 1994 29 3 185 195 10.1111/j.1600-0765.1994.tb01212.x 7515960

[B23] Sculean A Berakdar M Pahl S Windisch P Brecx M Reich E Donos N Patterns of cytokeratin expression in monkey and human periodontium following regenerative and conventional periodontal surgery J Periodontal Res 2001 36 4 260 268 10.1034/j.1600-0765.2001.036004260.x 11519700

[B24] Jiang Q Li D Cytokeratin expression in human junctional epithelium, oral epithelium and sulcular epithelium] Zhonghua Kou Qiang Yi Xue Za Zhi = Zhonghua Kouqiang Yixue Zazhi = Chin J Stomatol 2005 40 4 298 16191371

[B25] Kjörell U Östberg Y Virtanen I Thornell L-E Immunohistochemical analyses of autoimmune sialadenitis in man J Oral Pathol Med 1988 17 8 374 380 10.1111/j.1600-0714.1988.tb01300.x 2464679

[B26] Tavakoli M Bateni E Attarbashi-Moghadam F Talebi A Yaghini J Mogharehabed A Comparison of fibronectin in human marginal gingiva and interdental papilla using immunohistochemistry Dent Res J 2011 8 Suppl1 S109 PMC3556296 23372588

[B27] Bampton JL Shirlaw PJ Topley S Weller P Wilton JM Human junctional epithelium: demonstration of a new marker, its growth in vitro and characterization by lectin reactivity and keratin expression J Investig Dermatol 1991 96 5 708 717 10.1111/1523-1747.ep12470948 1708796

[B28] Abe Y Hara Y Sakua T Kato I Immunohistological study of cytokeratin 19 expression in regenerated junctional epithelium of rats J Periodontal Res 1994 29 6 418 420 10.1111/j.1600-0765.1994.tb01243.x 7533212

[B29] Feghall-Assaly M Sawaf M Ouhayoun J In situ hybridization study of cytokeratin 4, 13, 16 and 19 mRNAs in human developing junctional epithelium Eur J Oral Sci 1997 105 6 599 608 10.1111/j.1600-0722.1997.tb00224.x 9469612

[B30] Barrett A Cort E Patel P Berkovitz B An immunohistological study of cytokeratin 20 in human and mammalian oral epithelium Arch Oral Biol 2000 45 10 879 887 10.1016/S0003-9969(00)00050-9 10973561

[B31] Lu Q Samaranayake LP Darveau RP Jin L Expression of human β-defensin-3 in gingival epithelia J Periodontal Res 2005 40 6 474 481 10.1111/j.1600-0765.2005.00827.x 16302926

[B32] Salonen J Santti R An attempt to simulate junctional epithelium of human gingiva in vitro J Periodontal Res 1983 18 3 311 317 10.1111/j.1600-0765.1983.tb00365.x 6225859

